# Phosphorus and Heavy Metals Removal from Stormwater Runoff Using Granulated Industrial Waste for Retrofitting Catch Basins

**DOI:** 10.3390/molecules27217169

**Published:** 2022-10-23

**Authors:** Viravid Na Nagara, Dibyendu Sarkar, Rupali Datta

**Affiliations:** 1 Department of Civil, Environmental and Ocean Engineering, Stevens Institute of Technology, Hoboken, NJ 07030, USA; 2 Department of Biological Sciences, Michigan Technological University, Houghton, MI 49931, USA

**Keywords:** adsorbent, granules, waste-to-resource, water treatment, nutrient, heavy metals, retrofitting, green technology, stormwater runoff

## Abstract

Phosphorus and heavy metals are washed off and transported with stormwater runoff to nearby surface water bodies resulting in environmental and human health risks. Catch basins remain one of the primary gateways through which stormwater runoff and pollutants from urban areas are transported. Retrofitting catch basins to enhance their phosphorus and heavy metal removal can be an effective approach. In this study, aluminum-based water treatment residual (WTR, a non-hazardous byproduct of the water treatment process) was granulated via a green method to serve as a sustainable filter material, called WTR granules, for enhancing the capabilities of catch basins to remove phosphorus and heavy metals. The WTR granules were field tested in a parking lot in Hoboken, New Jersey. Twelve storm events were monitored. The results showed that the WTR granules significantly (*p* < 0.05) reduced dissolved P, Cu, and Zn, as well as total P, Cu, Pb, and Zn concentrations in stormwater runoff without signs of disintegration. No flooding or water ponding was observed during the implementation. Results suggest the WTR granules are an inexpensive, green filter material that can be used for retrofitting catch basins to remove phosphorus and heavy metals effectively.

## 1. Introduction

Nutrients and heavy metals commonly generated in urban areas are washed off by stormwater runoff. Major sources of nutrients include fertilizers, soil erosion, deposition of combustion products on impervious surfaces, and biogenic materials (e.g., grass clippings and leaf litter) [1,2]. Heavy metals are typically released from vehicular activities (e.g., tire and brake wear, degradation of asphalt road surfaces, exhaust emissions, etc.), roadway maintenance operations, roof runoff, and atmospheric deposition [1,3,4]. Phosphorus (P), copper (Cu), lead (Pb), and zinc (Zn) are among the most commonly found pollutants in stormwater runoff [1,5,6]. These pollutants are transported by stormwater runoff either bound to particles or in a dissolved form. Particulate-bound pollutants are associated with suspended particulates in stormwater [7]. The partitioning between the soluble and particle-bound phases largely depends on the pH and redox conditions [5,6].

Rainwater contains various chemicals resulting from atmospheric pollution [8]. When rainwater accumulates on surfaces, it becomes stormwater runoff and picks up pollutants that are deposited on the surfaces. This pollutant cocktail is transported through stormwater systems and discharged to nearby surface water generally without treatment. Due to excessive amounts of pollutants, such as nutrients and heavy metals, stormwater runoff is considered a major source of water quality impairment resulting in contamination of water and sediments in receiving water bodies [2,9]. Excess nutrients can cause major ecological impacts, such as eutrophication, harmful algal blooms, and fish kills [10]. Heavy metals are of concern due to their high toxicity, biomagnification, and non-biodegradability. They are toxic for many aquatic organisms even at trace levels [5]. From a human health perspective, exposure to lead can cause brain damage and irreversible neurological and behavioral effects. Despite being essential trace elements for human health, continuous exposure to Cu and Zn at excessive levels may cause adverse health effects such as kidney damage, cholesterol problems, anemia, and even death [11].

Although many stormwater control measures (e.g., rain gardens, bioswales, pervious pavements, green roofs, retention ponds, detention basins) have been developed and are available for implementation, they may not be suitable due to their design, construction costs, or the limited space available in many urban areas [12]. Stormwater runoff is still conveyed by conventional stormwater networks by draining through catch basins [13]. However, catch basins have very limited capabilities to remove stormwater pollutants [14]. They are primarily designed for retaining suspended solids, and these accumulated solids were found to serve as a reservoir for pollutants, such as pesticides, and eventually cause downstream contamination [13]. Since catch basins are universal in most urban areas, retrofitting existing catch basins is a promising approach to combat stormwater pollution due to its simplicity and having no additional requirement for land allocation.

Catch basin inserts (CBIs) are typically employed to retrofit catch basins. They are devices that can be either mounted inside or attached to the stormwater grates of catch basins to capture incoming stormwater pollutants [15]. Generally, CBIs are made with a geotextile or a permeable container to capture gross pollutants [16,17]. However, these designs are not effective in removing other pollutants, such as nutrients and heavy metals, especially in dissolved form. Incorporating filter media in stormwater infrastructures, such as catch basins, can enhance their pollutant removal from stormwater runoff, typically through filtration and adsorption (including ion-exchange, surface precipitation, and complexation) [18,19,20,21,22]. Although different filter media are commercially available, incorporating them into the CBIs can be cost-prohibitive [22]. Therefore, it is necessary to develop economical filter media with high pollutant removal capability that have a minimal environmental impact to serve as a green technology for stormwater treatment.

To tackle this challenge, aluminum-based water treatment residuals (WTR) were selected for developing this green technology. WTR is a byproduct of the water treatment process where aluminum salts are used as primary coagulants [23]. In many countries, WTR is generated at a rate of more than 300,000 tons per year, which is expected to increase significantly in the coming years [24]. Disposal of WTRs is a major challenge, due to the scarcity of landfill space and the high cost of disposal. Hence, diverting WTR from landfills and utilizing it as a raw material for other purposes is increasingly being explored [25]. WTR is a non-hazardous material capable of removing various environmental pollutants, including P, Cu, Pb, and Zn, which are common stormwater pollutants [23,26,27].

Since the primary purpose of catch basins is to drain excess stormwater runoff from impervious areas, it is critical to ensure that the hydraulic capacity of the catch basins is not significantly hindered and that the risk of flooding or water pooling is minimized. However, the major disadvantage of using WTR for this purpose is its intrinsically low hydraulic conductivity. In our previous study, WTR was utilized as an adsorbent media for retrofitting catch basins. To enhance the permeability of the filter material, other coarse materials, including sand and carbon material, were mixed with WTR. However, this strategy involved a compromise between hydraulic conductivity and removal performance, since WTR with a relatively higher removal capability was replaced by sand and carbon material, which had relatively lower removal capabilities [22].

To overcome this tradeoff, WTR was granulated to produce WTR granules by following a patented method described in the U.S. Patent Application Publication No. US 2020/0316556 A1 [27]. With this method, organic materials and WTR are processed through a simple and low-energy procedure. The advantages of the WTR granules are (1) the production cost of the WTR granules is low since the raw materials involve waste and organic materials which can be acquired at low cost, (2) two major environmental issues, water quality, and waste management, are addressed at the same time, (3) the WTR granules are easy to emplace and maintain as they can be retrofitted in the catch basins and replenished during typical catch basin inspection and maintenance, and (4) the WTR granules are versatile and can be deployed in various water treatment or stormwater systems. Since this WTR granulation method helps to reduce waste, does not involve the release of hazardous substances, and has a low energy requirement, the resulting product, WTR granules, can be considered a green filter material from a green chemistry perspective [28,29].

In our previous studies, the physicochemical characteristics and pollutant removal performance of the WTR granules were investigated on a lab scale [27,30]. This current study serves as a scaled-up field demonstration of this green technology. The objectives of this study were to (i) evaluate the performance of WTR granules for use as CBI retrofit to remove P, Cu, Pb, and Zn from stormwater runoff under field conditions; (ii) evaluate the disintegration potential of WTR granules during field implementation; (iii) investigate whether the spent WTR granules can be disposed of as non-hazardous waste; and (iv) estimate the lifespan of the WTR granules.

## 2. Materials and Methods

### 2.1. Reagents and Materials

WTR was provided by the New Jersey American Water (NJAW) Water Treatment Plant in Bridgewater Township, NJ, where aluminum salts were the main coagulant. Acetic acid (Glacial, Certified ACS) and nitric acid (TraceMetal™ Grade) were procured from Sigma-Aldrich (St. Louis, MO, USA) and Fisher Scientific (Fair Lawn, NJ, USA), respectively. Deionized (DI) water was used for chemical preparation and analyses throughout the study. Certified reference solutions were supplied by High-Purity Standards (North Charleston, SC, USA). Potassium alginate and eggshell powder were purchased from BOC Sciences (Shirley, NY, USA) and Natural Innovative Solutions (Albany, OR, USA), respectively.

### 2.2. WTR Granule Generation

The patented method as described in the U.S. Patent Application Publication No. US 2020/0316556 A1 [27] was followed for generating WTR granules. Briefly, after being obtained from the drinking water treatment plant, the WTR was air-dried, ground, and sieved through a 1 mm sieve. To further reduce the particle size for increasing surface area, the <1 mm fraction of WTR was milled with a planetary ball mill (Fritsch Pulverisette 5, Fritsch GmbH, Idar-Oberstein, Germany) at 590 rpm (grinding jar rotating) and 290 rpm (tray rotating) for 18 min. Then, the milled WTR was added to 2% (*w/v*) potassium alginate solution to achieve 15% milled WTR (*w/v*). After thoroughly mixing with an overhead stirrer, the WTR-alginate solution was added dropwise to a calcium solution, which was prepared by adding eggshell powder at 6% (*w/v*) into 10% (*v/v*) acetic solution. After soaking in the calcium solution, the granules were washed several times with DI water until no residue from the calcium solution was observed. Then, the WTR granules were air-dried and kept in polyethylene resealable bags in a dry place until further use.

### 2.3. Study Area

**Figure 1:**
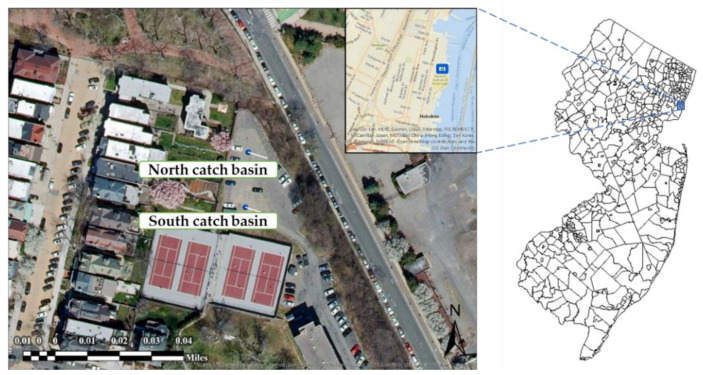
Aerial photo of the two catch basins at the Stevens CPH parking lots, Hoboken, NJ, USA.

The monitoring location of this study was Castle Point Hall (CPH) parking lot at Stevens Institute of Technology, Hoboken, NJ, USA (Figure 1). This location was selected based on the relatively high background concentrations of phosphorus and heavy metals in stormwater runoff observed during preliminary monitoring. The retrofitting was carried out in two catch basins located in the northern section of this parking lot, which received stormwater runoff from the parking area, tennis courts, sloping vegetated areas, and two large dumpsters located upstream.

### 2.4. Experimental Design

**Figure 2:**
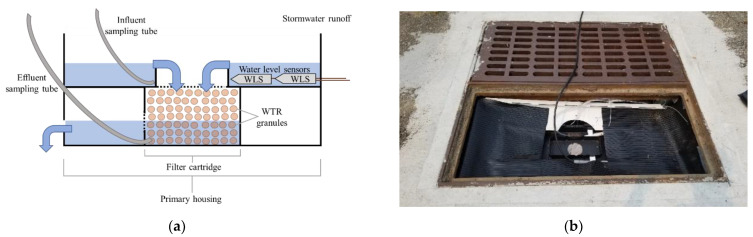
(**a**) Diagram of the catch basin inserts, and (**b**) catch basin insert installed in the site.

To retrofit the catch basins with the WTR granules, a CBI was required to house the WTR granules in each catch basin. In addition to serving as filter cartridges, the CBIs must allow the sampling of influent (pretreated runoff) and effluent (treated runoff) for performance evaluation. Therefore, two custom-made CBIs were designed and built. For each catch basin, a two-tier plastic toolbox was used as the primary housing of the CBI (Figure 2). The top tier was used as an influent reservoir to collect influent before passing through the filter cartridge loaded with the WTR granules which was installed in the bottom tier. Sampling tubes were placed in the influent reservoir and at the outlet of the filter cartridge for influent and effluent sample collection.

### 2.5. Sample Collection and Analytical Methodology

At each catch basin, two autosamplers (ISCO Model 6712c portable samplers with submerged probe flow module, Teledyne ISCO, Lincoln, NE, USA) were installed. One autosampler was for influent collection, while the other autosampler was for effluent collection. The sampling mechanism was triggered when the water level in the influent reservoir reached approximately 2 cm. The sampling containers were acid-washed and rinsed with DI water before being used for sample collection. To minimize cross-contamination between sampling attempts, the autosamplers underwent a purging procedure before and after each sampling attempt.

After each storm event, the samples were retrieved to the analytical lab as soon as the weather conditions permitted within 24 h. The samples were measured for pH and EC using portable meters. Total suspended solids (TSS) concentrations were determined in accordance with Standard Method 2540D [31]. In addition, an aliquot of each sample was digested with HNO_3_ by following the U.S. EPA method 200.7 [32] for total element concentration analysis, whereas another aliquot of each sample was filtered through 0.45-µm nylon syringe filters and acidified with HNO_3_ for dissolved element concentration analysis. The total and dissolved concentrations of phosphorus and heavy metals were determined using an inductively coupled plasma–optical emission spectrometer (ICP-OES, 5100 Agilent Technologies, Santa Clara, CA, USA). For quality control purposes, a field blank was created by pouring DI water into an additional cleaned bottle in each autosampler and analyzed along with the samples to assess whether any contamination was introduced. At the end of the study, the spent WTR granules in the catch basin inserts were subjected to toxicity characteristic leaching procedure (TCLP) test by following the EPA SW-846 Methods 1311 [33].

### 2.6. Statistical Analysis

The values of the measured water quality parameters within a storm event from each catch basin were calculated as event mean concentrations (EMCs), which represent the average concentration of a single runoff event. Although the primary goal of stormwater treatment should be focused on the effluent water quality, which indicates the subsequent ecological impacts of the discharge, the influent water quality should be taken into account to evaluate the contribution and effectiveness of the retrofit. Therefore, the treatment effectiveness was presented based on two parameters.

**Figure 3:**
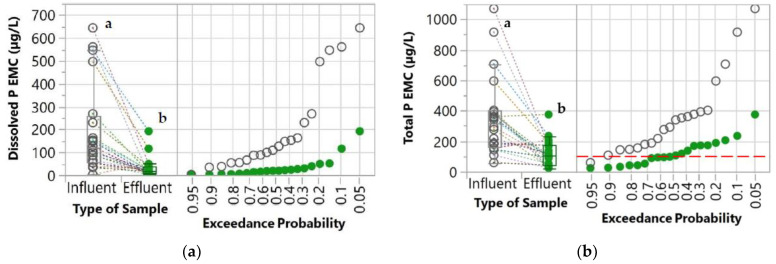
Box plot and normal probability plot of (**a**) dissolved and (**b**) total P EMCs of influent and effluent samples. A pair of influent and effluent EMCs from the same storm event and catch basin are connected with dotted lines. Different lowercase letters (a and b) indicate significant differences between influent and effluent P content (*p* < 0.05). The red horizontal dashed line represents water quality goals [34].

**Figure 4:**
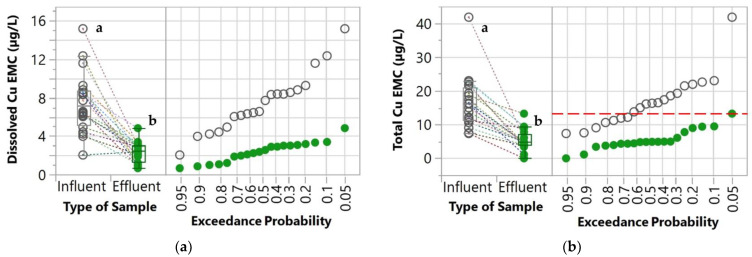

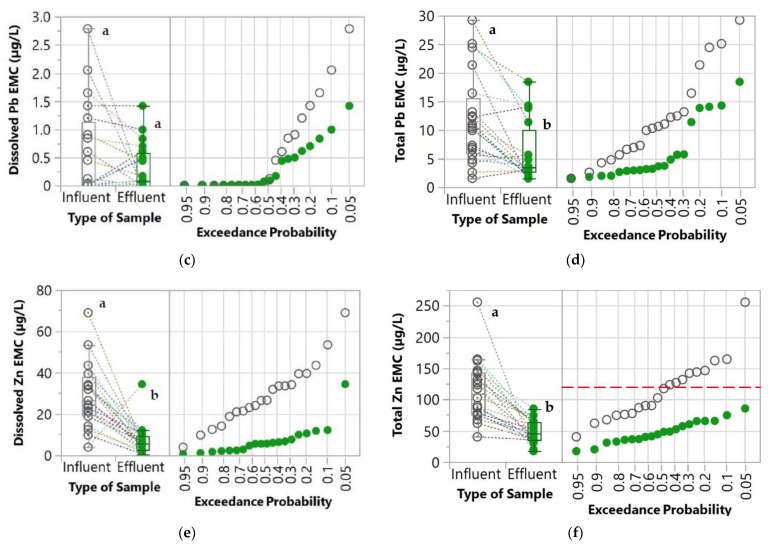
Box plot and normal probability plot of (**a**) dissolved Cu, (**b**) total Cu, (**c**) dissolved Pb, (**d**) total Pb, (**e**) dissolved Zn, and (**f**) total Zn EMCs of both influent and effluent samples. A pair of influent and effluent EMCs from the same storm event and catch basin are connected with dotted lines. Different lowercase letters (a and b) indicate significant differences between influent and effluent (*p* < 0.05). The red horizontal dashed line represents water quality goals [34]. Note: The water quality goal for Pb (65 µg/L) is not shown since it is greater than the upper limit of the *y*-axis.

**Figure 5:**
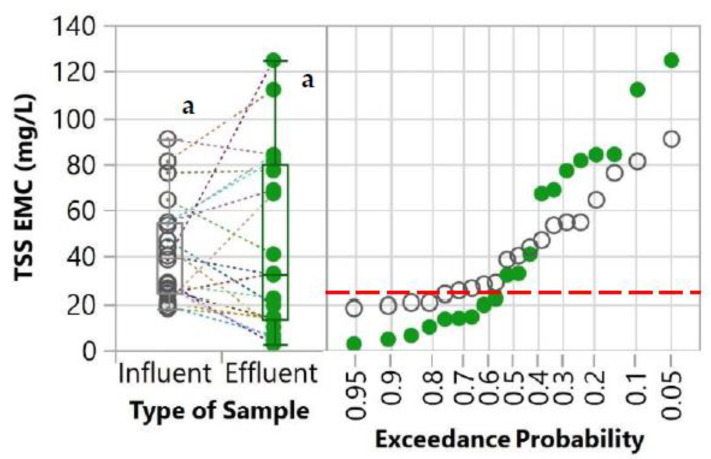
Box plot and normal probability plot of TSS EMC of both influent and effluent. A pair of influent and effluent EMCs from the same storm event and catch basin are connected with dot lines. Different lowercase letters (a and b) indicate significant differences between influent and effluent (*p* < 0.05). The red horizontal dashed line represents water quality goals [34].

**Figure 6:**
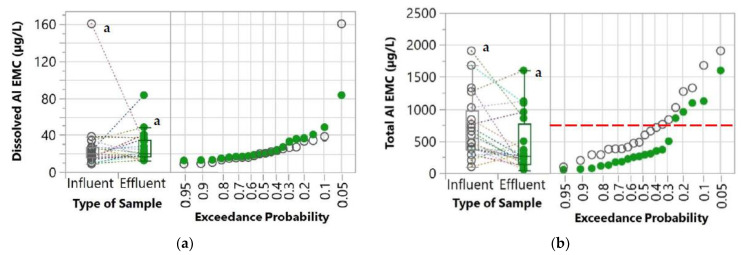
Box plot and normal probability plot of (**a**) dissolved and (**b**) total Al EMCs of both influent and effluent. A pair of influent and effluent EMCs from the same storm event and catch basin are connected with dotted lines. Different lowercase letters (a and b) indicate significant differences between influent and effluent (*p* < 0.05) samples. The red horizontal *dashed* line represents water quality goals [34].

The first parameter evaluated was the percent exceedance probabilities of the influent and effluent concentrations which were determined by comparing the values with the water quality goals. These water quality goals were derived from the United States Environmental Protection Agency’s (USEPA) National Recommended Water Quality Criteria [34]. These goals were also used in other studies for evaluating the performance of stormwater control measures [35,36]. Since the water quality criteria are established for total concentrations, only the total concentrations of the pollutants were compared with these goals. For TSS, the New Jersey Surface Water Quality Standard [37] was used as a water quality goal because the USEPA’s National Recommended Water Quality Criteria was not available for TSS. These water quality goals are indicated in Table 1 and Figure 3, Figure 4, Figure 5 and Figure 6.

The second parameter evaluated was the percent change between influent and effluent water quality parameters, which is calculated as:

(1) $$\%\;\mathrm{Change}=\frac{\mathrm{Median}\;\mathrm{effluent}\;\mathrm{EMC}\;-\mathrm{Median}\;\mathrm{influent}\;\mathrm{EMC}}{\mathrm{Median}\;\mathrm{influent}\;\mathrm{EMC}}\times 100$$

The median values were selected for calculating percent change instead of mean values to minimize the influence of intrinsically high variations in the water quality parameters in stormwater. For each water quality parameter, a % change with a positive value indicates an increase in the water quality parameter in the effluent, compared to the influent, while a negative value indicates a decrease.

All statistical analyses were conducted in the JMP statistical software package (JMP 15.2, SAS Institute Inc., Cary, NC, USA). Principal Component Analysis (PCA) is a dimension-reduction approach, which is often used for analyzing high-dimensional data, involving multiple variables, such as for quantifying the water quality index [38]. In this study, PCA was employed to identify the correlations between pollutant removal and environmental parameters. The Tukey’s honestly significant difference (HSD) test was performed to determine significant differences (*p* < 0.05) in the water quality parameters between influent and effluent.

### 2.7. Lifespan Analysis

The lifespan of a filter system is a crucial factor for large-scale implementation, especially the design and maintenance of the treatment system. Some adsorbents are designed to be reusable for multiple adsorption–desorption cycles. In those cases, regeneration of adsorbents is considered for determining the lifespan of the adsorbents. However, regeneration is generally achieved through solvent or thermal treatment to desorb the adsorbed pollutants from the adsorbent surfaces [39,40,41]. Therefore, additional energy and/or chemicals are required, which translates into additional cost and complexity in operation and maintenance, making it less optimal from a green technology standpoint.

Our intention was to develop this adsorbent as an inexpensive, simple, green technology. Using WTR as a primary sorbent enables this technology to be economically and technically feasible for one-time use. Therefore, the lifespan of the WTR granules was estimated based on one adsorption. The lifespan can be determined based on the sorption capacity, which was determined in the previous study [27], and the quantity of the filter material, as well as the stormwater pollutant concentrations and the volume of stormwater. According to the equation adopted from Yan et al. (2016), the lifespan of the WTR granules can be calculated as [42]:

(2) $$Lifespan=\frac{q_{max}\times D_{WTRG}\times \rho_{WTRG}\;}{D_{precipitation}\times C}\times f$$

where $q_{max}$ = Langmuir maximum sorption capacity,$\;D_{WTRG}$ = depth of the WTR granules loaded in the catch basin inserts (3 in), $\rho_{WTRG}$ = bulk density of the WTR granules (1.25 kg/L), $D_{precipitation}$ = average annual precipitation (49.5 in), C = median inlet concentration from the field monitoring, and f = catchment area/filter area ratios (0.0005).

The Langmuir maximum sorption capacities of the WTR granules for P and Zn were 5 and 10 mg/g, respectively [27]. Since Cu and Pb sorption isotherm data were poorly fitted by the Langmuir model (R^2^ < 0.39), and the concentration of Cu and Pb was found in relatively low concentrations in the field, Cu and Pb were not considered for this analysis. The median inlet concentrations of dissolved P and dissolved Zn in the field were 118.32 and 26.64 µg/L, respectively. Because adsorption involves the removal of dissolved pollutants, only the dissolved concentrations were considered for this analysis. The average annual precipitation of 49.5 in. was determined from the 10-years precipitation record at the Jersey City station, which is the closest station to the field site [43]. The other parameters were determined based on the experimental design.

## 3. Results and Discussion

In total, 12 storm events were monitored at the Stevens CPH parking lot (Figure 1) over five months (Table 1). Samples were collected from both catch basins, except for storm events nos. 7, 10, 11, and 12, when the samples were collected only from the north catch basin. This was because the autosamplers at the south catch basin were not functioning properly during storm event no. 7. For storm events nos. 10, 11, and 12, the autosamplers at the south catch basin could not be deployed due to the presence of a trailer at the site. The residual concentrations of P, Cu, Pb, and Zn in the field blanks were negligible, indicating no contamination of samples occurred. The results of the water quality monitoring are listed in Table 2 and Table 3. Hydraulically, no flooding or water ponding was observed during the monitoring period, indicating no negative impact of WTR granules on the hydraulic performance of the catch basin.

### 3.1. Phosphorus

The WTR granules consistently and effectively removed both dissolved and total P. The dissolved and total P EMCs of influent and effluent are presented as a box plot and normal probability plot in Figure 3. The effluent P EMCs were lower than the influent P EMCs in most of the storm events as indicated by the declining slope of the dotted lines in the box plots, which connect the influent and effluent P EMCs from the same storm event and catch basin. According to the statistical analysis, the effluent P EMCs were significantly lower (*p* < 0.05) than the influent P EMCs for both dissolved and total concentrations. As shown in Table 2, the median P EMCs in the effluent were lower than in the influent by 83.2% (dissolved) and 67.6% (total). By considering the water quality goal, the WTR granules substantially reduced the likelihood of total P exceedance from 91.3% to 51.3% (Figure 3b).

Removal of P by the WTR granules was primarily attributed to the inherent P removal capability of the WTR, which has been evaluated in previous studies [44,45,46,47,48]. Total P removal is generally controlled by filtration of the particulate-bound phase and adsorption of the dissolved phase. Based on the results, the difference between the median influent and effluent P EMCs of total concentration (213.88 µg/L) is substantially higher than that of dissolved concentration (98.41 µg/L), indicating that the removal of P involved both filtration and adsorption mechanisms. WTR primarily consisted of aluminum hydroxide (AlOH) [30,49,50], which is highly reactive. Since P occurs as phosphate in aquatic systems, P adsorption on WTR occurs by OH^-^ replacement, resulting in the formation of an inner sphere complex [45]. In addition, the physical properties of WTR (e.g., porosity and specific surface area) are also favorable for P removal [23].

With the sustainable granulation method [27], the removal potential of WTR had been enabled for minimizing P in stormwater runoff, which otherwise flows into the catch basins and is discharged to surface water without treatment.

### 3.2. Heavy Metals

Removal of Cu, Pb, and Zn by the WTR retrofit was consistently observed for both dissolved and total concentrations, indicated by the decreasing slope of the dotted lines in both box plots (Figure 4). When the influent EMC was relatively low, the decreasing trend was not observed. The inconsistent removal at lower concentrations was expected and in agreement with our previous field monitoring [22] and other stormwater treatment studies in the literature [51,52]. It is likely that when the influent concentrations are very low, it is difficult to achieve further reduction. Therefore, the effluent concentrations may be equal to or higher than the influent concentrations, resulting in negative removal. These very low concentrations are typically referred to as irreducible concentrations. The concept of irreducible concentrations in stormwater treatment has been recognized for decades [53]. Although negative removal is not desirable, the increased effluent concentrations resulting from these irreducible concentrations were well below the water quality goals and rarely occurred. Therefore, the negative ecological impact was not of concern.

The effluent EMCs of the three heavy metals were significantly lower (*p* < 0.05) than the influent EMCs for both dissolved and total concentrations, except for dissolved Pb which had relatively low influent concentrations (<3 µg/L) throughout the monitoring period. Pb concentrations in stormwater have been reducing substantially in the past decades [6]. This is associated with the phasing out of leaded gasoline and the substitution of Pb in other sources such as Pb-based paints and car parts (e.g., brake linings, tires, and weights added to vehicles for tire balance) [6]. For Pb removal, the median influent EMC of the total Pb concentrations was 67.3% lower than that of the influent. For Cu removal, the median effluent EMC was lower than the median influent EMC by 65.7% and 70.0% for dissolved and total concentrations, respectively. For Zn removal, the median removal efficiencies of dissolved and total Zn EMCs were 78.9% and 58.0%, respectively.

The results show that the WTR granules used in this study were more effective in removing heavy metals in stormwater compared to the previous approach [22]. In the previous study, WTR was amended with sand and placed over a layer of carbon material to improve permeability and was then field tested in a parking lot to evaluate its performance in removing dissolved Cu, Pb, and Zn [22]. By comparing the median removal efficiencies from this study with the previous study, the removal performance of the WTR granules, the new design, was substantially improved. The median removal efficiencies of dissolved Cu increased from 27.4% to 65.7%, and from 69.3% to 78.9% for dissolved Zn removal. The superior removal by the WTR granules compared to the previous design was attributable to the higher content of WTR in the filter media. This was because the filter material was primarily composed of WTR, whereas in the previous version the WTR was mixed with sand and carbon material, which had relatively lower pollutant removal capabilities [22].

Significant reduction in both dissolved and total concentrations of the heavy metals (except for dissolved Pb) suggested that the removal of heavy metals involved both filtration of the particulate-bound phase and adsorption of the dissolved phase. Pb was found predominantly in the particulate-bound phase, while Cu and Zn were relatively higher in dissolved fractions, which is consistent with previous studies [6,54]. The removal of dissolved heavy metals by WTR was dominated by surface complexation via proton exchange [55].

By considering the water quality goals, the WTR granules substantially reduced the likelihood of total Cu exceedance from 64.1% to only 5.0%, and of total Zn exceedance from 45.5% to 0%. Therefore, the deployment of the WTR granules can help to improve water quality and reduce the environmental risk from heavy metal contamination.

### 3.3. Total Suspended Solids (TSS)

A box plot and a normal probability plot of TSS EMCs of the influent and effluent samples are shown in Figure 5. Although the reduction of TSS in the effluent was not statistically significant compared to the influent, the median TSS EMC in the effluent (32 mg/L) was lower than in the influent (40 mg/L). By comparing with the water quality goal, the exceedance of the TSS is reduced from 72.6% in the influent to 55.7% in the effluent.

TSS is an important water quality parameter, which reflects the transport of pollutants and ecological productivity [56]. Although TSS could represent total metal concentrations in stormwater runoff to a certain extent, the reduction of TSS does not necessarily translate to a reduction in total metal concentrations [57]. This is because pollutants are adsorbed to different particle size fractions unproportionally, and the partitioning of pollutants between dissolved and particulate-bound phases is highly influenced by site-specific conditions [57,58]. This was evident in this study as we also observed the discrepancy between the significant reduction of the total pollutant EMCs and no significant difference between influent and effluent TSS EMCs.

In addition to stormwater pollution, TSS also represents solid contents in stormwater runoff that can accumulate in the stormwater treatment systems and adversely impact their long-term hydraulic performance. The risk of hydraulic failure (e.g., clogging) is particularly high for fine-grained filter media, such as soil- and clay-based filter media, since a clogging layer will form over the top of the filter media preventing stormwater runoff to flow through and be treated [59].

Suspended solids in stormwater runoff are generally removed through sedimentation and/or physical filtration [60,61,62]. Sedimentation is typically used for the removal of coarse particles and often requires a long retention time, while physical filtration is generally achieved by passing runoff through a layer of fine filter media [17,62]. Minimal to no suspended solids removal by the retrofits in this study was expected since the WTR granules were relatively coarse (diameter of approximately 2–5 mm) and they were not integrated with any mesh or fabric, such as non-woven geotextile, to serve as a filter layer in the flow path. Therefore, the risk of clogging was low because of minimal solids accumulation. On the other hand, the comparable TSS EMC between influent and effluent demonstrated that the WTR granules did not adversely contribute to suspended solids transport (i.e., the WTR granules did not serve as a source of particulate pollutants). For future trials, the retrofit could be equipped with non-woven geotextile to further enhance suspended solids removal.

### 3.4. Potential of Aluminum Export

It is critical to ensure that the WTR granules served as pollutant sinks without pollutant export. Although no significant increase in the effluent TSS was observed compared to the influent, the key composition of the WTR granules in the stormwater was monitored to ensure that the WTR granules did not disintegrate and contribute to pollutant export. Since the WTR used in this study was primarily comprised of aluminum, Al concentrations were also monitored throughout the study period. Dissolved and total Al EMCs of both influent and effluent samples are shown in Figure 6. The difference between Al EMCs in the influent and effluent samples was minimal and not statistically significant (*p* < 0.05) for both dissolved and total concentrations. The median influent and effluent Al EMC values were 20.66 µg/L and 20.06 µg/L, respectively, for dissolved concentrations, and 538.66 µg/L and 275.43 µg/L, for total concentrations. By comparing with the water quality goal, the WTR granules substantially reduced the likelihood of total Al exceedance from 34% to 25.2%. The result shows there was no Al export or sign of WTR granules disintegration. In contrast, a slight removal of total Al was found.

### 3.5. Effect of Environmental Parameters

Environmental parameters (e.g., background electrolyte, pH, rainfall intensity, total rainfall, and temperature) have been reported to impact pollutants in stormwater runoff and their removal [6,30,63,64]. These elements could be influential factors leading to variations in pollutant removal observed during the monitoring period. To further investigate the influence of the environmental parameters on pollutant removal, PCA was conducted for the identification of correlations between the % change of the pollutants and other environmental parameters, including influent EC, influent pH, average rainfall intensity, total rainfall, and temperature. Influent EC and pH were selected over effluent EC and pH because they represent the background conditions of the site. Rainfall and temperature data were acquired from North American Land Data Assimilation System (NLDAS) Hourly NOAH data, site name X407-Y125 [65], using CUAHSI HydroDesktop [66]. This data site had the closest proximity to the study area compared to other data sites. It is located near Liberty State Park, NJ, which is adjacent to the Hudson River, as is our study area. Although the value obtained from this dataset may not be as accurate as direct measurements in the study area, the obtained data were sufficient to provide relative meteorological conditions for this evaluation.

**Figure 7:**
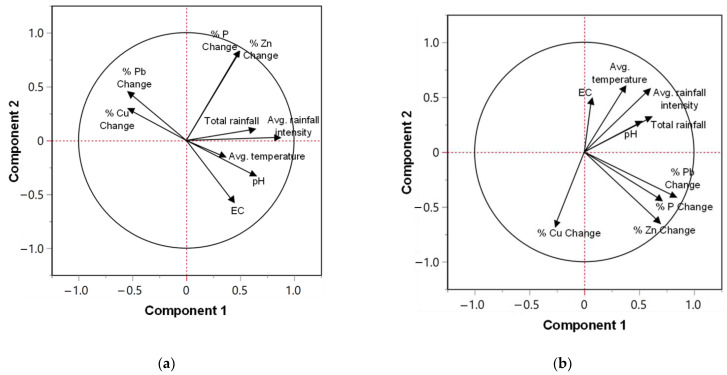
Principal component loading plots for (**a**) dissolved concentrations and (**b**) total concentrations.

The correlation between individual parameters can be determined from the angle between loading vectors. A smaller angle between the loading vectors of two parameters indicates a higher correlation between the two parameters, whereas an angle of 180 degrees indicates an inverse correlation. Orthogonal vectors represent uncorrelated variables. As shown in Figure 7a, % Change of dissolved Cu and Pb are inversely correlated with average temperature, EC, and pH. In other words, dissolved Cu and Pb removal increased as EC and pH increased. However, the average temperature had relatively less influence, as indicated by the short length of its loading vector. Dissolved P and Zn removal was found to be minimally influenced by the three environmental parameters as indicated by the orthogonal vectors. Total rainfall and rainfall intensity were found to have a limited influence on dissolved pollutant removal. In terms of total concentrations, the environmental parameters were not correlated with Pb, Zn, and P removal, except for Cu removal (Figure 7b).

**Figure 8:**
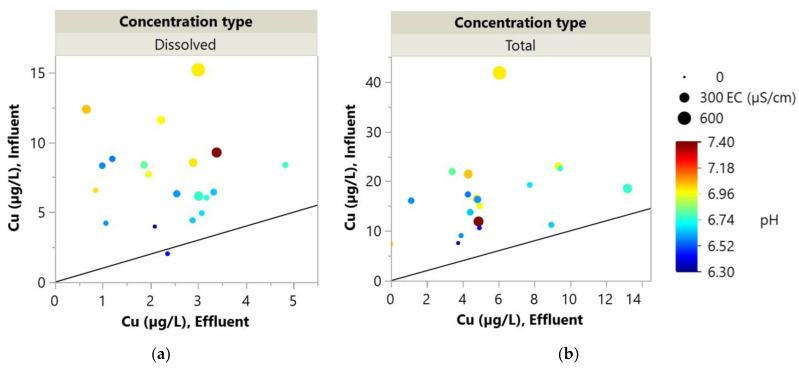
Bubble plots of (**a**) dissolved and (**b**) total Cu EMCs of both influent and effluent. The size of the bubbles represents the corresponding EC, while colors represent the corresponding pH. The 1:1 lines are included for reference, representing no change in the concentrations.

Different correlations between % Change and the environmental parameters for dissolved and total were observed. Since Pb was found in relatively low concentrations in this area, the results of Cu, which also has a strong inverse correlation with the environmental parameters, are presented as bubble plots in Figure 8. These plots were included to illustrate the different effects of the environmental parameters on dissolved and total concentrations as indicated by the PCA. The 1:1 lines in Figure 8 represent no change in the concentrations. The upper region of the line represents the desirable area where the effluent EMCs were lower than the influent EMCs, while the lower region of the line represents the negative removal area where the effluent EMCs were higher than the influent EMCs. In the case of dissolved Cu EMCs (Figure 8a), the gradient of color and size of the bubbles are apparent. As the bubbles are located further from the 1:1 line, the size of the bubbles increases, and the color of the bubbles changes from cool colors to hot colors. In contrast, such a trend was not observed in the case of total Cu EMCs (Figure 8b). This was because dissolved pollutants were likely to be removed primarily through adsorption mechanisms, which could be more sensitive to environmental parameters compared to total concentrations that include particulate-bound pollutants. The pH and temperature dependence of dissolved Cu and Pb removal by WTR was consistent with the previously reported study by Duan and Fedler (2021) [67].

### 3.6. Disposal of the Spent WTR Granules

Disposal and regeneration are important factors determining the feasibility of implementing adsorbent systems, especially in large-scale operations [68]. Many filter materials can be regenerated through chemical or thermal regeneration processes [39,68], which could be chemical- or energy-intensive. Since the WTR granules are a low-cost adsorbent material, direct disposal as a typical solid waste is preferred to minimize the cost and environmental footprint of regeneration processes. To determine whether the spent WTR granules can be disposed of as a non-hazardous waste, the TCLP test, which mimics a typical municipal landfill condition, was conducted to evaluate chemical leaching from the spent WTR granules. From the TCLP test, the leached concentrations of the Resource Conservation and Recovery Act (RCRA) metals were all below the hazardous waste toxicity characteristic criteria, as shown in Table 4. This result shows that the release of undesired chemicals under landfilling conditions is not a concern and that the spent WTR granules can be disposed of as a non-hazardous waste.

### 3.7. Lifespan Analysis

Based on the analysis discussed in Section 2.7, the filter system’s lifespan for P and Zn removal is estimated to be 1.6 and 14 years, respectively. Therefore, the more conservative estimate of 1.6 years can be used to determine the typical lifespan of WTR granules at this site. It is worth noting that lifespan estimation depends on site-specific parameters. The results obtained from this analysis were for illustrative purposes. Prior to implementation, it is recommended that the influent concentrations at the target locations should be monitored so that the appropriate amount of WTR granules needed could be determined. For example, in locations where influent concentrations are high, the depth of WTR granules can be increased accordingly to achieve the desired lifespan. Since catch basins should be inspected at least annually [14], a lifespan of at least one year is recommended. Hence, no additional maintenance schedule for granule replenishment would be required.

## 4. Conclusions

Results indicate good performance of the WTR granules in removing P, Cu, Pb, and Zn from stormwater runoff under real-world conditions without exporting Al to the stormwater runoff. No signs of flow hindrances, such as flooding or water ponding, by the WTR granules were observed throughout the study. The spent WTR granules can be disposed of as non-hazardous waste in municipal landfills. The WTR granules can be easily deployed in catch basins by personnel who are familiar with stormwater systems, without requiring additional training. Due to the simple implementation and low procurement and disposal costs, WTR granules are a cost-effective and green technology that can be used for retrofitting existing catch basins to remove phosphorus and heavy metals. It is worth noting that this study focused on the pollutant removal performance of the WTR granules. Future research should focus on hydraulic and long-term seasonal performance to further broaden our understanding of the potential of WTR granules in P and heavy metal removal. Subsequently, the design for field implementation needs to be further optimized.

## Figures and Tables

**Table 1:** Storm events monitored at the Stevens CPH parking lot.

Storm Event No.	Date	Storm Event No.	Date
1	19 August 2021	7	16 October 2021
2	21 August 2021	8	26 October 2021
3	1 September 2021	9	29 October 2021
4	14 September 2021	10	12 November 2021
5	23 September 2021	11	1 January 2022
6	5 October 2021	12	17 January 2022

**Table 2:** Results of the dissolved and total concentrations of phosphorus and heavy metals.

Pollutant	Statistics	Dissolved Concentration				Total Concentrations						
		Inf. ^a^ (µg/L)	Eff. ^b^ (µg/L)	Change (%)		Inf. ^a^ (µg/L)	Eff. ^b^ (µg/L)	Change (%)		Water Quality Goal ^c^ (µg/L)	Exceedance (%)	
											Inf. ^a^	Eff. ^b^
P	Median	118.32	19.91	−83.2		316.26	102.38	−67.6		100	91.3	51.3
	Mean	198.98	33.72			364.18	124.07					
	Std Dev	198.53	45.4			268.66	86.98					
Cu	Median	7.14	2.45	−65.7		16.19	4.86	−70.0		13	64.1	5.0
	Mean	7.48	2.37			16.65	5.47					
	Std Dev	3.11	1.06			7.66	3.02					
Pb	Median	0.08	0.08	0		10.46	3.42	−67.3		65	0	0
	Mean	0.6	0.32			11.8	6.03					
	Std Dev	0.83	0.42			7.84	5.22					
Zn	Median	26.64	5.63	−78.9		109.88	46.13	−58.0		120	45.5	0
	Mean	29.05	7.04			113.99	47.83					
	Std Dev	15.37	7.34			48.51	17.93					
Al	Median	20.66	20.06	−2.9		538.66	275.43	−48.9		750	34	25.2
	Mean	27.96	26.7			706.26	447.61					
	Std Dev	32.34	16.77			498.49	435.78					
TSS	Median	-	-	-		40 ^d^	32 ^d^	−18.1		25 ^d^	72.6	55.7
	Mean	-	-			43 ^d^	46 ^d^					
	Std Dev	-	-			22 ^d^	38 ^d^					

^a^ Influent. ^b^ Effluent. ^c^ Water quality goals were derived from National Recommended Water Quality Criteria by USEPA [34]. ^d^ Unit: mg/L.

**Table 3:** Results of the pH and electrical conductivity.

Parameter	Unit	Statistics	Influent	Effluent	% Change
pH	-	Median	6.77	6.69	−1.18
		Mean	6.79	6.69	
		Std Dev	0.26	0.21	
EC	µS/cm	Median	99.97	88.18	−11.79
		Mean	142.77	104.13	
		Std Dev	131.15	43.18	

**Table 4:** Toxicity characteristic leaching values of metals and metalloids measured in spent WTR granules.

Analyte	Spent WTR Granules (µg/L)	USEPA Regulatory Level (µg/L) ^a^
Ag	0.40	5000
As	16.21	5000
Ba	48.98	100,000
Cd	0.60	1000
Cr	0.72	5000
Hg	BDL ^b^	200
Pb	11.24	5000
Se	10.66	1000
Al	10,476	NR ^c^

^a^ TCLP criterion: maximum concentrations of contaminants for the toxic characteristics from Title 40 CFR 261.24—Toxicity characteristic; ^b^ BDL—Below detection limit; ^c^ NR—Not regulated under Title 40 CFR 261.24—Toxicity characteristic. No regulatory level is set by USEPA.

## Data Availability

Data presented in this study are available on request from the corresponding author.

## Sample Availability

Not available.

## References

[1] Müller A., Österlund H., Marsalek J., Viklander M. (2020). The Pollution Conveyed by Urban Runoff: A Review of Sources. Sci. Total Environ..

[2] Yang Y.-Y., Lusk M.G. (2018). Nutrients in Urban Stormwater Runoff: Current State of the Science and Potential Mitigation Options. Curr. Pollut. Reports.

[3] McKenzie E.R., Money J.E., Green P.G., Young T.M. (2009). Metals Associated with Stormwater-Relevant Brake and Tire Samples. Sci. Total Environ..

[4] Sörme L., Lagerkvist R. (2002). Sources of Heavy Metals in Urban Wastewater in Stockholm. Sci. Total Environ..

[5] Pamuru S.T., Forgione E., Croft K., Kjellerup B.V., Davis A.P. (2022). Chemical Characterization of Urban Stormwater: Traditional and Emerging Contaminants. Sci. Total Environ..

[6] Huber M., Welker A., Helmreich B. (2016). Critical Review of Heavy Metal Pollution of Traffic Area Runoff: Occurrence, Influencing Factors, and Partitioning. Sci. Total Environ..

[7] Han Y.H., Lau S.L., Kayhanian M., Stenstrom M.K. (2006). Correlation Analysis among Highway Stormwater Pollutants and Characteristics. Water Sci. Technol..

[8] Anil I., Alagha O., Blaisi N.I., Mohamed I.A., Barghouthi M.H., Manzar M.S. (2019). Source Identification of Episodic Rain Pollutants by New Approach: Combining Satellite Observations and Backward Air Mass Trajectories. Aerosol Air Qual. Res..

[9] Mohiuddin K.M., Ogawa Y., Zakir H.M., Otomo K., Shikazono N. (2011). Heavy Metals Contamination in Water and Sediments of an Urban River in a Developing Country. Int. J. Environ. Sci. Technol..

[10] Conley D.J., Paerl H.W., Howarth R.W., Boesch D.F., Seitzinger S.P., Havens K.E., Lancelot C., Likens G.E. (2009). Controlling Eutrophication: Nitrogen and Phosphorus. Science.

[11] Sharma S. (2015). Heavy Metals in Water.

[12] Davis A.P., Shokouhian M., Sharma H., Minami C., Winogradoff D. (2003). Water Quality Improvement through Bioretention: Lead, Copper, and Zinc Removal. Water Environ. Res..

[13] Sy N.D., Wheeler S.S., Reed M., Haas-stapleton E., Reyes T., Bear-johnson M., Kluh S., Cummings R.F., Su T., Xiong Y. (2022). Pyrethroid Insecticides in Urban Catch Basins: A Potential Secondary Contamination Source for Urban Aquatic Systems ☆. Environ. Pollut..

[14] USEPA (1999). Stormwater O&M Fact Sheet: Catch Basin Cleaning.

[15] Alam M.Z., Anwar A.H.M.F., Heitz A., Sarker D.C. (2018). Improving Stormwater Quality at Source Using Catch Basin Inserts. J. Environ. Manage..

[16] Alam M.Z., Anwar A.H.M.F., Sarker D.C., Heitz A., Rothleitner C. (2017). Characterising Stormwater Gross Pollutants Captured in Catch Basin Inserts. Sci. Total Environ..

[17] Alam M.Z., Anwar A.H.M.F., Heitz A. (2018). Stormwater Solids Removal Characteristics of a Catch Basin Insert Using Geotextile. Sci. Total Environ..

[18] Okaikue-Woodi F.E.K., Cherukumilli K., Ray J.R. (2020). A Critical Review of Contaminant Removal by Conventional and Emerging Media for Urban Stormwater Treatment in the United States. Water Res..

[19] Huber M., Hilbig H., Badenberg S.C., Fassnacht J., Drewes J.E., Helmreich B. (2016). Heavy Metal Removal Mechanisms of Sorptive Filter Materials for Road Runoff Treatment and Remobilization under De-Icing Salt Applications. Water Res..

[20] Tran H.N., You S.J., Hosseini-Bandegharaei A., Chao H.P. (2017). Mistakes and Inconsistencies Regarding Adsorption of Contaminants from Aqueous Solutions: A Critical Review. Water Res..

[21] Drapper D., Hornbuckle A. (2015). Field Evaluation of a Stormwater Treatment Train with Pit Baskets and Filter Media Cartridges in Southeast Queensland. Water.

[22] Na Nagara V., Sarkar D., Barrett K., Datta R. (2021). Greening the Gray Infrastructure: Green Adsorbent Media for Catch Basin Inserts to Remove Stormwater Pollutants. Environ. Technol. Innov..

[23] Makris K.C., Harris W.G., O’Connor G.A., Obreza T.A., Elliott H.A. (2005). Physicochemical Properties Related to Long-Term Phosphorus Retention by Drinking-Water Treatment Residuals. Environ. Sci. Technol..

[24] Zhao Y., Liu R., Awe O.W., Yang Y., Shen C. (2018). Acceptability of Land Application of Alum-Based Water Treatment Residuals—An Explicit and Comprehensive Review. Chem. Eng. J..

[25] Turner T., Wheeler R., Stone A., Oliver I. (2019). Potential Alternative Reuse Pathways for Water Treatment Residuals: Remaining Barriers and Questions—A Review. Water. Air. Soil Pollut..

[26] Xu D., Lee L.Y., Lim Y., Lyu Z., Zhu H., Ong S.L., Hu J. (2020). Water Treatment Residual: A Critical Review of Its Applications on Pollutant Removal from Stormwater Runoff and Future Perspectives. J. Environ. Manage..

[27] Sarkar D., Na Nagara V., Datta R. (2020). Method for Generating a Granular, Green Sorbent Media for Filtration of Contaminated Water by Processing Aluminum-Based Drinking Water Treatment Residuals. U.S. Patent.

[28] Ghernaout D., Ghernaout B., Naceur M.W. (2011). Embodying the Chemical Water Treatment in the Green Chemistry-A Review. Desalination.

[29] Anastas P.T., Warner J.C. (1998). Green Chemistry: Theory and Practice.

[30] Na Nagara V., Sarkar D., Elzinga E.J., Datta R. (2022). Removal of Heavy Metals from Stormwater Runoff Using Granulated Drinking Water Treatment Residuals. Environ. Technol. Innov..

[31] Baird R.B., Eaton A.D., Rice E.W., Bridgewater L. (2017). 2540 D. Total Suspended Solids Dried at 103–105 °C. Standard Methods for the Examination of Water & Wastewater.

[32] USEPA (2001). Method 200.7: Trace Elements in Water, Solids, and Biosolids by Inductively Coupled Plasma-Atomic Emission Spectrometry.

[33] USEPA (1992). Method 1311: Toxicity Characteristic Leaching Procedure.

[34] USEPA (2004). National Recommended Water Quality Criteria.

[35] Stagge J.H., Davis A.P., Jamil E., Kim H. (2012). Performance of Grass Swales for Improving Water Quality from Highway Runoff. Water Res..

[36] Li H., Davis A.P., Asce F. (2009). Water Quality Improvement through Reductions of Pollutant Loads Using Bioretention. J. Environ. Eng..

[37] NJDEP (2020). New Jersey Administrative Code 7:9B Surface Water Quality Standards.

[38] Manzar M.S., Benaafi M., Costache R., Alagha O., Mu’azu N.D., Zubair M., Abdullahi J., Abba S.I. (2022). New Generation Neurocomputing Learning Coupled with a Hybrid Neuro-Fuzzy Model for Quantifying Water Quality Index Variable: A Case Study from Saudi Arabia. Ecol. Inform..

[39] Dutta T., Kim T., Vellingiri K., Tsang D.C.W., Shon J.R., Kim K.H., Kumar S. (2019). Recycling and Regeneration of Carbonaceous and Porous Materials through Thermal or Solvent Treatment. Chem. Eng. J..

[40] Lata S., Singh P.K., Samadder S.R. (2015). Regeneration of Adsorbents and Recovery of Heavy Metals: A Review. Int. J. Environ. Sci. Technol..

[41] Miguet M., Goetz V., Plantard G., Jaeger Y. (2016). Sustainable Thermal Regeneration of Spent Activated Carbons by Solar Energy: Application to Water Treatment. Ind. Eng. Chem. Res..

[42] Yan Q., Davis A.P., Asce F., James B.R. (2016). Enhanced Organic Phosphorus Sorption from Urban Stormwater Using Modified Bioretention Media: Batch Studies. J. Environ. Eng..

[43] NJDEP NJDEP New Jersey Department of Environmental Protection-Rainfall. https://njdep.rutgers.edu/rainfall/graph_basic.php?station_id=.

[44] Babatunde A.O., Zhao Y.Q., Yang Y., Kearney P. (2008). Reuse of Dewatered Aluminium-Coagulated Water Treatment Residual to Immobilize Phosphorus: Batch and Column Trials Using a Condensed Phosphate. Chem. Eng. J..

[45] Ippolito J.A., Barbarick K.A., Heil D.M., Chandler J.P., Redente E.F. (2003). Phosphorus Retention Mechanisms of a Water Treatment Residual. J. Environ. Qual..

[46] Makris K.C., Harris W.G., O’Conno G.A., Obreza T.A. (2004). Phosphorus Immobilization in Micropores of Drinking-Water Treatment Residuals: Implications for Long-Term Stability. Environ. Sci. Technol..

[47] Makris K.C., Sarkar D., Salazar J., Punamiya P., Datta R. (2010). Alternative Amendment for Soluble Phosphorus Removal from Poultry Litter. Environ. Sci. Pollut. Res..

[48] Soleimanifar H., Deng Y., Wu L., Sarkar D. (2016). Water Treatment Residual (WTR)-Coated Wood Mulch for Alleviation of Toxic Metals and Phosphorus from Polluted Urban Stormwater Runoff. Chemosphere.

[49] Punamiya P., Sarkar D., Rakshit S., Datta R. (2013). Effectiveness of Aluminum-Based Drinking Water Treatment Residuals as a Novel Sorbent to Remove Tetracyclines from Aqueous Medium. J. Environ. Qual..

[50] Nagar R., Sarkar D., Makris K.C., Datta R. (2010). Effect of Solution Chemistry on Arsenic Sorption by Fe- and Al-Based Drinking-Water Treatment Residuals. Chemosphere.

[51] Borne K.E., Fassman E.A., Tanner C.C. (2013). Floating Treatment Wetland Retrofit to Improve Stormwater Pond Performance for Suspended Solids, Copper and Zinc. Ecol. Eng..

[52] Thompson J., Schwartz J.S., Hathaway J.M. (2020). Performance Evaluation of a Regenerative Stormwater Conveyance System: Case Study in Knoxville, Tennessee. J. Environ. Eng..

[53] Schueler T. (1996). Irreducible Pollutant Concentrations Discharged from Stormwater Practices. Watershed Prot. Technol..

[54] Lange K., Österlund H., Viklander M., Blecken G.T. (2020). Metal Speciation in Stormwater Bioretention: Removal of Particulate, Colloidal and Truly Dissolved Metals. Sci. Total Environ..

[55] Schulthess C.P., Huang C.P. (1990). Adsorption of Heavy Metals by Silicon and Aluminum Oxide Surfaces on Clay Minerals. Soil Sci. Soc. Am. J..

[56] Rügner H., Schwientek M., Beckingham B., Kuch B., Grathwohl P. (2013). Turbidity as a Proxy for Total Suspended Solids (TSS) and Particle Facilitated Pollutant Transport in Catchments. Environ. Earth Sci..

[57] Baum P., Kuch B., Dittmer U. (2021). Adsorption of Metals to Particles in Urban Stormwater Runoff—Does Size Really Matter?. Water.

[58] Andral M.C., Roger S., Montréjaud-Vignoles M., Herremans L. (1999). Particle Size Distribution and Hydrodynamic Characteristics of Solid Matter Carried by Runoff from Motorways. Water Environ. Res..

[59] Hatt B.E., Fletcher T.D., Deletic A. (2008). Hydraulic and Pollutant Removal Performance of Fine Media Stormwater Filtration Systems. Environ. Sci. Technol..

[60] Rodak C.M., Moore T.L., David R., Jayakaran A.D., Vogel J.R. (2019). Urban Stormwater Characterization, Control, and Treatment. Water Environ. Res..

[61] Sansalone J., Kuang X., Ying G., Ranieri V. (2012). Filtration and Clogging of Permeable Pavement Loaded by Urban Drainage. Water Res..

[62] Nyström F., Nordqvist K., Herrmann I., Hedström A., Viklander M. (2020). Removal of Metals and Hydrocarbons from Stormwater Using Coagulation and Flocculation. Water Res..

[63] Yang H., Dick W.A., Mccoy E.L., Phelan P.L., Grewal P.S. (2013). Field Evaluation of a New Biphasic Rain Garden for Stormwater Flow Management and Pollutant Removal. Ecol. Eng..

[64] Maniquiz M.C., Lee S., Kim L.-H. (2010). Multiple Linear Regression Models of Urban Runoff Pollutant Load and Event Mean Concentration Considering Rainfall Variables. J. Environ. Sci..

[65] Mitchell K.E., Lohmann D., Houser P.R., Wood E.F., Schaake J.C., Robock A., Cosgrove B.A., Sheffield J., Duan Q., Luo L. (2004). The Multi-Institution North American Land Data Assimilation System (NLDAS): Utilizing Multiple GCIP Products and Partners in a Continental Distributed Hydrological Modeling System. J. Geophys. Res. D Atmos..

[66] Ames D.P., Horsburgh J.S., Cao Y., Kadlec J., Whiteaker T., Valentine D. (2012). HydroDesktop: Web Services-Based Software for Hydrologic Data Discovery, Download, Visualization, and Analysis. Environ. Model. Softw..

[67] Duan R., Fedler C.B. (2021). Adsorptive Removal of Pb^2+^ and Cu^2+^ from Stormwater by Using Water Treatment Residuals. Urban Water J..

[68] Singh N.B., Nagpal G., Agrawal S., Rachna (2018). Water Purification by Using Adsorbents: A Review. Environ. Technol. Innov..

